# A Chinese SCA36 pedigree analysis of *NOP56* expansion region based on long-read sequencing

**DOI:** 10.3389/fgene.2023.1110307

**Published:** 2023-03-27

**Authors:** Jinlong Zou, Fengyu Wang, Zhenping Gong, Runrun Wang, Shuai Chen, Haohan Zhang, Ruihua Sun, Chenhao Gao, Wei Li, Junkui Shang, Jiewen Zhang

**Affiliations:** ^1^ Department of Neurology, Henan University People’s Hospital, Henan Provincial People’s Hospital, Zhengzhou, China; ^2^ Department of Neurology, Henan Provincial People’s Hospital, Zhengzhou University People’s Hospital, Zhengzhou, China; ^3^ Department of Neurology, Xinxiang Medical University, Henan Provincial People’s Hospital, Zhengzhou, China; ^4^ Academy of Medical Sciences, Zhengzhou University, Zhengzhou, Henan, China

**Keywords:** SMRT sequencing, NOP56 gene, GGCCTG, spinocerebellar ataxia, repeat interruptions

## Abstract

**Introduction:** Spinocerebellar ataxias 36 (SCA36) is the neurodegenerative disease caused by the GGCCTG Hexanucleotide repeat expansions in *NOP56*, which is too long to sequence using short-read sequencing. Single molecule real time (SMRT) sequencing can sequence across disease-causing repeat expansion. We report the first long-read sequencing data across the expansion region in SCA36.

**Methods:** We collected and described the clinical manifestations and imaging features of Han Chinese pedigree with three generations of SCA36. Also, we focused on structural variation analysis for intron 1 of the *NOP56* gene by SMRT sequencing in the assembled genome.

**Results:** The main clinical features of this pedigree are late-onset ataxia symptoms, with a presymptomatic presence of affective and sleep disorders. In addition, the results of SMRT sequencing showed the specific repeat expansion region and demonstrated that the region was not composed of single GGCCTG hexanucleotides and there were random interruptions.

**Discussion:** We extended the phenotypic spectrum of SCA36. We applied SMRT sequencing to reveal the correlation between genotype and phenotype of SCA36. Our findings indicated that long-read sequencing is well suited to characterize known repeat expansion.

## Introduction

Spinocerebellar ataxia type 36 (SCA36) (OMIM: 614153) is a spinal cerebellar ataxia disease first identified in Japan and Spain. (Kobayashi et al., 2011; Garcia-Murias et al., 2012) The incidence of the disease is high in East Asia (Japan and China) and Spain. Sporadic cases have also been reported in Poland, the United State and France. (Sugihara et al., 2012; Valera et al., 2017) The frequency of SCA36 in autosomal dominant ataxia in mainland China is about 1.6%, and in sporadic spinal cerebellar ataxia (SCA), it is about 0.32%, accounting for 3.5% of SCA families in Japan and 6.3% of SCA families in Spain. (Garcia-Murias, et al., 2012; Sugihara, et al., 2012; Zeng et al., 2016) It is mainly characterized by a late-onset, slowly progressive cerebellar syndrome typically involving motor neurons or associated with hearing loss. (Kobayashi, et al., 2011; Garcia-Murias, et al., 2012; Ikeda et al., 2012) Cognitive and affective disorders have also been reported. (Abe et al., 2012) The brain Magnetic resonance image (MRI) of SCA36 patients with asymptomatic and pre-ataxia showed atrophy of the upper cerebellar vermis early in the ataxia phase and diffuse cerebellar atrophy years later in the course of the disease. (Aguiar et al., 2017; Xie et al., 2022) The pathology is characterized by a neuronal loss in the Purkinje cell layer of the cerebellum and dentate nucleus. In addition to the diffused cerebellar atrophy, the loss of motor neurons in the hypoglossal nucleus and anterior horn of the upper cervical cord has also been reported. (Ikeda, et al., 2012) SCA36 is caused by the expansion of GGCCTG hexanucleotide repeats in the first intron in the nucleolar protein 56 (*NOP56*) gene on 20p13. (Kobayashi, et al., 2011) The normal alleles contained 3–14 repeats, but the expanded alleles usually contained between 650 and 2,500 repeats. Also, it is reported that the small repeat number of 25, 30, and 31 could cause this disease. (Obayashi et al., 2015)

The high GC content and long repeat motifs are characteristic of GGCCTG hexanucleotide repeat expansion. The repeat expansion sequence was extremely long approximately 3,990–15,000 base. Therefore, the use of conventional diagnostic methods is limited. Currently, repeat-primed polymerase chain reaction (RP-PCR) screening combined with Southern blotting is commonly used to diagnose the disease. RP-PCR is a fragment analysis method based on the Sanger platform which has high sensitivity and specificity. However, because the expansion size is beyond the limits of analysis by conventional fragment analysis, the specific fragment size and repeat number can’t be accurately determined. (Ishige et al., 2012) Traditionally, repeat number can be estimated by Southern blotting, which is a labor-intensive and radioactive method. Therefore, because specific nucleotide sequences across of the SCA36 repeat expansion could not be detected, we tried to find new methods to solve these difficulties.

Single molecule real time (SMRT) sequencing is a third-generation sequencing technology with a long-read length, high accuracy, uniform coverage, no PCR amplification and no GC preference. (Eid et al., 2009) SMRT has been widely used for genome assembly and disease diagnosis. (Ardui et al., 2018) For example, the telomere-to-telomere (T2T) consortium has applied this technology for CHM13 genome sequence assembly, as well as for detecting mutations causing for facioscapulohumeral muscular dystrophy (FSHD), SCA10, and other diseases. (McFarland et al., 2015; Dai et al., 2020; Gershman et al., 2022) Due to the pathogenic region of intron 1 of the *NOP56* gene with high GC content and extremely long sequence, SMRT sequencing has good application in the diagnosis and research of SCA36.

In this study, we collected and compiled a Han Chinese family pedigree with three generations of SCA36, and then described the clinical manifestations and imaging features. We applied SMRT technology to detect and analyze the base composition of repeat expansion sequences to explore the correlation between genotypes and phenotypes. Further, we believe that these findings can deepen our knowledge of SCA36 and may lay the foundation for revealing the genetic mechanism, diagnosis, and treatment of this rare disease.

## Materials and methods

### Participants

The study subjects were members of three generations of a Han Chinese ataxia family pedigree in Henan province. A detailed medical history and physical examination record of the proband (II_5_) and some family members (II_7_, II_9_, II_11_, III_13,_ and III_15_) were evaluated by two experienced neurologists. Peripheral blood specimens were obtained from the proband (II_5_) and some members (II_7,_ II_9_, II_11_, II_13_ II_15_, III_4_, and III_10_) for genetic testing. This study was approved by the local ethics committee, and all patients signed an informed consent form.

### Clinical features

Clinical physical examinations and evaluations were performed in the proband (II_5_) and other members (II_9_, II_11_, and II_15_), including the Scale for Assessment and Rating of Ataxia (SARA),Mini-Mental State Examination (MMSE), Montreal Cognitive Assessment (MoCA), Hamilton Anxiety Scale (HAMA), Hamilton Depression Scale (HAMD) and Pittsburgh Sleep Quality Index (PSQI). Electrophysiological examinations conducted include; nerve conduction velocity (NCV), electromyography (EMG), motor evoked potential (MEP), somatosensory evoked potential (SSEP), visual evoked potential (VEP), brainstem auditory response (BAEP) and pure-tone audiometry (PTA). However, II_7_ and II_13_ received only clinical history questioning, physical examination, and scale assessment.

### Magnetic resonance images acquisition and preprocessing

The proband (II_5_) and three family members (II_9_, II_11_, and II_15_) underwent a detailed MRI examination. Among them, II_7_ underwent MRI flat-scan examination before one month, and the image acquisition was performed using Siemens Magnetom Prisma 3.0T MRI scanner in the following sequence: T1WI, T2WI, FLAIR, DWI, 3D-T1, and DTI images.

3D-T1 images were segmented in the MNI space using a Brain Label. (Ye et al., 2018) The whole brain was segmented into different anatomical structures using a Brain Label with 283 optimal regions. Brain atlases were transformed into local space by aligning 3D-T1 images using a symmetric differential isomorphic image alignment algorithm built into ANTs. We quantitatively analyzed the segmented brain regions of each patient to obtain the volume of each brain region. Brain volume data were then compared with those of healthy individuals of the same sex and age according to the Brain Label database.

DTI image preprocessing was performed using the PANDA to generate FA DEC and AD maps. (Wasserthal et al., 2019) The automated fiber quantification analysis of white matter fibers was then carried out. The data obtained for FA values are expressed as mean ± standard deviation. SPSS 22.0 statistical software was applied to statistically analyze the measurements at different sites bilaterally and paired *t*-test was used for the bilateral comparisons. *p* < 0.05 was considered to be statistically significant.

## Genetic analysis

### Repeat-primed PCR

Due to the initial suspicion of SCA, the RP-PCR and capillary electrophoresis were performed to identify SCA subtypes of the proband (II_5_) and some members (II_7,_ II_9_, II_11_, II_13_, II_15_, III_4_, and III_10_), including SCA1, 2, 3, 6, 7, 8, 10, 12, 17, 36, and DRPLA. RP-PCR relies on repeat primers with amplified alleles that anneal to produce the results for PCR products separated by capillary electrophoresis is “ladder”. For DNA fragment analysis, RP-PCR products were analyzed using the ABI-Prism 3730XL Genetic Analyzer, and the data were analyzed using GeneMarker software.

### Exome sequencing

The proband (II_5_) and two other members (II_11_ and II_15_) were subjected the exome sequencing through Illumina HiSeq platform. Single nucleotide variants (SNV)and insertions and deletions (InDels) were analyzed by Genome Analysis Tool Kit (GATK) software. Single nucleotide variants (SNV) and insertions and deletions (InDels) were analyzed by GATK software. Genome Analysis Tool Kit Variants with minor allele frequencies >0.5% were filtered out by the databases, including the gnomAD, ExAC and 1000 genome databases. Functional prediction of candidate variants was performed using SIFT, Polyphen-2 and Mutation Taster software. All variants were interpreted according to ACMG recommendations based on the ClinVar, OMIM, and HGMD databases. Sanger sequencing was used to validate the genetic variants detected by exome sequencing.

### Long-read genome sequencing

We selected the three samples (II_5_, II_7_, and II_11_), which presented different clinical symptoms, and were sequenced by long-read genome sequencing. PacBio CLR sequencing has an error rate of 11%–15%. The sequencing errors are random and can be corrected by increasing the coverage of sequencing. To obtain more full-length subreads to get the exact repeat region length and interruptions landscape, we set the coverage sequencing to ×100.1. Genomic DNA Sample Preparation: Samples were collected, and high molecular weight genomic DNA was prepared by the CTAB method and followed by purification with QIAGEN^®^ Genomic kit (Cat#13343, QIAGEN) for regular sequencing, according to the standard operating procedure provided by the manufacturer. The DNA degradation and contamination of the extracted DNA was monitored on 1% agarose gels. DNA purity was then detected using NanoDrop™ One UVVis spectrophotometer (Thermo Fisher Scientific, USA), of which OD260/280 ranging from 1.8 to 2.0 and OD 260/230 is between 2.0 and 2.2. At last, DNA concentration was further measured by Qubit^®^ 4.0 Fluorometer (Invitrogen, USA). 2. Library preparation and sequencing: The SMRTbell Continuous Long Read (CLR) library was constructed for sequencing according to PacBio’s standard protocol (Pacific Biosciences, CA, USA) using either 10 kb or 20 kb preparation solutions. The main steps for library preparation are: 1) gDNA shearing, 2) DNA damage repair, end repair and A-tailing, 3) ligation with hairpin adapters from the SMRTbell Express Template Prep Kit 2.1 (Pacific Biosciences), 4) size selection, and 5) binding to polymerase. Briefly, a total amount of 5 μg DNA per sample was used for the DNA library preparations. The genomic DNA sample was sheared by g-TUBEs (Covaris, USA) according to the expected size of the fragments for the library. Single-strand overhangs were then removed, and DNA fragments were damage repaired, end repaired and A-tailing. Then the fragments ligated with the hairpin adaptor for PacBio sequencing. Target fragments were screened by the BluePippin (Sage Science, USA). The SMRTbell library was then purified by AMPure PB beads, and Agilent 2,100 Bioanalyzer (Agilent technologies, USA) was used to detect the size of library fragments. Sequencing was performed on a PacBio Sequel II instrument with Sequencing Primer V4 and Sequel II Binding Kit 2.1 in Haorui Genomics. After sequencing, we take the effective filtering strategies to improve the sequencing data quality. The filtered reads were assembled into the individual genome using NextDenove software. Then, we mainly analyzed the GGCCTG repeat region in the intron 1 region of the *NOP56* gene. To better observe the structural variation, the genome GRCh38 was artificially modified by inserting d (GGCCTG)_1000_ before the original d (GGCCTG)_4_ to construct a fake GRCh38 sequence. The subreads were then compared to GRCh38 and fake GRCh38. The GGCCTG repeat number in each sample were counted, and the number of subreads containing GGCCTG repeat number greater than 650 and the full-length subreads of GGCCTG repeat number and length were also analyzed. Based on other about SCA studies, except for core motif GGCCTG, the pathogenic regions exist interruption motifs in similar diseases, such as SCA10, so it is may likely that not all regions actually obtained are composed of GGCCTG tandem repeats. (Matsuura et al., 2006; McFarland et al., 2013) In order to visualize the nucleotide sequence, the schematic representation of the motifs was produced based on the Practical Extraction and Report Language. Statistical analysis of the tandem repeat motifs in the d (GGCCTG) n region by the schematic representation of all full-length subreads were performed to determine the distribution regularity of the motifs.

## Result

### Clinical presentation

**FIGURE 1:**
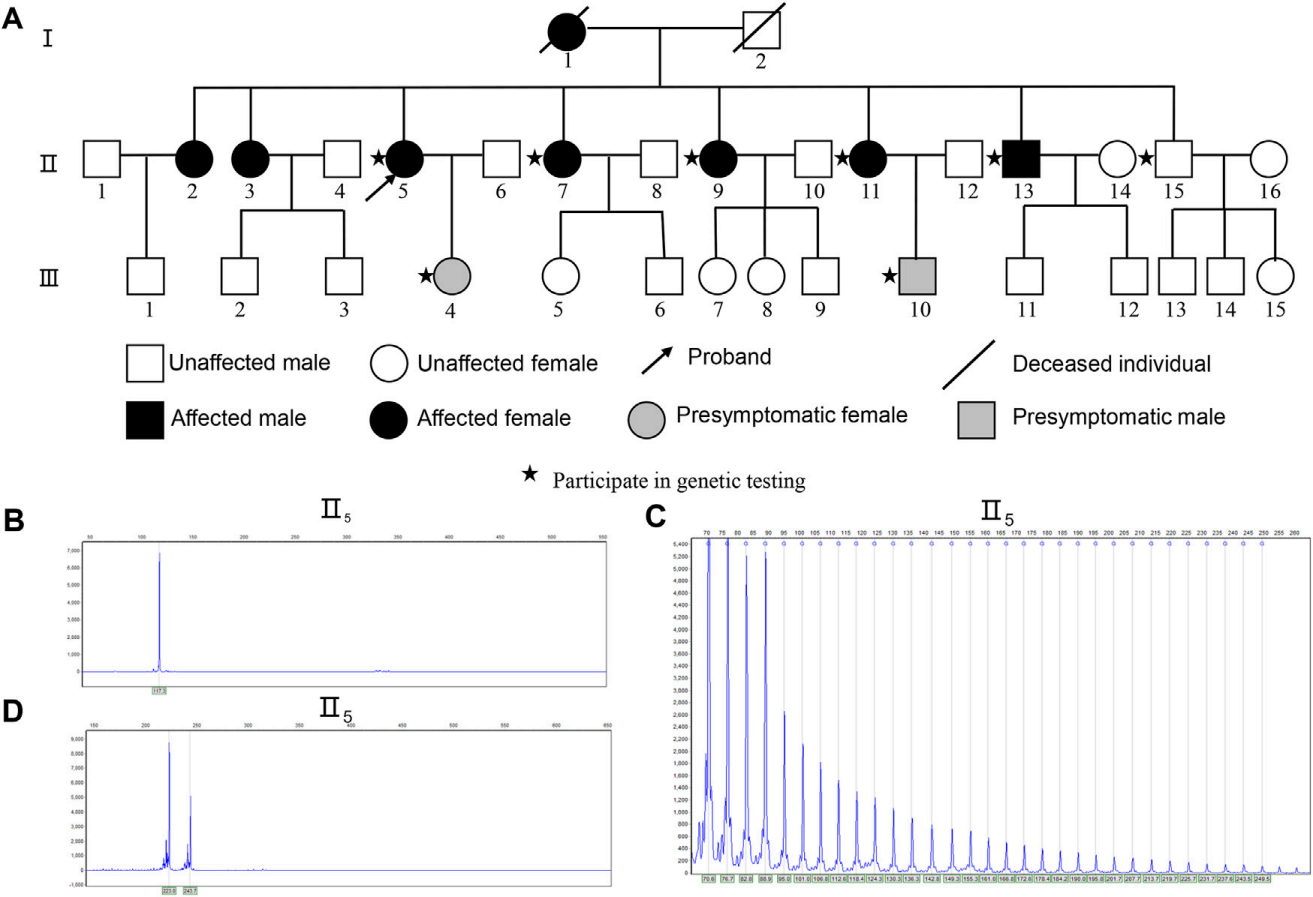
Identification of Expanded GGCCTG Repeat within *NOP56* in the SCA family pedigree. **(A)** Genealogical structure of the family with spinocerebellar ataxia. The outco me of RP-PCR about II_5_ **(B)** The conventional PCR of II_5_ showed only one peak indicated homozygous or with a significant expansion. **(C)** II_5_ showed a characteristic ladder pattern with a 6-bp periodicity indicated repeat expansion in *NOP56*. **(D)** The conventional PCR of outcome II_5_ shows the CAG repeat number is 41 times in *TBP* gene, Sanger sequencing shows the CAG repeat number is 43.

The family pedigree of ataxia is shown in (Figure 1). 6 patients and 2 presymptomatic individuals were identified in the pedigree, and the detailed data of each affected member are shown in Table 1. The proband (II_5_) was a 62-year-old female. At age 40, this patient was observed to have affective disorders manifested by low motivation and irritability, along with severe insomnia and long-term use of valium. At age 47, the patient began experiencing unstable walking. At age 49, her speech became increasingly incoherent, and she occasionally choked on water, along with self-perceived hearing loss and inability to clearly hear what others said. At age 58, she developed blurred vision, occasional dizziness, and memory loss. The patient was 62 years old at the time of evaluation and showed cognitive impairment (MMSE: 22/30); (MoCA: 12/30), with mild depression, anxiety and sleep disturbances.

**TABLE 1:** The Clinical features of patients.

	II-5	II-7	II-9	II-11	II-13	II-15
Gender	F	F	F	F	M	M
Age at onset	47	46	50	42	42	40
Age at examination	63	56	52	49	44	42
Truncal ataxia	++	++	+	+	+	-
Limb ataxia	+	+	±	+	+	-
Dysarthria	++	+++	-	+	-	-
Blurred vision	+	+	-	+	-	-
Nystagmus	-	-	-	-	-	-
Limitation of gaze	-	-	-	-	-	-
Hearing loss	++	NA	+	-	-	-
Hyperreflexia	++	+	-	+	-	-
Babinski sign	++	+	-	+	+	-
Cognitive impairment	+	+	-	-	-	-
Tongue atrophy	-	+	-	-	-	-
Tongue fasciculation	-	+	-	-	-	-
Muscle atrophy (limbs and trunk)	-	-	-	-	-	-
Muscle fasciculation (Limbs and trunk)	-	+	-	-	-	-
Epilepsy	-	-	-	-	-	+
SARA score	10	8.5	3	8.5	4	1
MMSE score	22	23	28	28	28	28
MOCA score	12	17	26	25	26	19
HAMA score	19	8	10	20	19	5
HAMD score	18	4	5	25	20	3
PSQI score	17	16	16	19	16	7
PTA	M-SNHL	NA	M-SNHL	-	NA	-
NCV	-	NA	-	PNI	NA	-
EMG	-	NA	-	NI	NA	-
MEP	PTCA-L	NA	-	PTCA-B	NA	-
SSEP	-	NA	-	-	NA	-
VEP	-	NA	-	-	NA	-
BAEP	APPI-B	NA	-	-	NA	-
	APCI-R					

Abbreviations: –, normal; +, mild, ++, moderate; NA, data not available; SARA, scale for assessment and rating of ataxia; MMSE, mini mental state examination; MOCA, montreal cognitive assessment; PTA, pure tone audiometry; M-SNHL, mild sensorineural hearing loss; NCV, never conduction velocity; PNI, peripheral nerve impairment; EMG, electromyogram; NI, neurogenic impairment; MEP, motor evoked potential; PTCA-L, pyramidal tract conduction abnormalities in the left lower extremity; PTCA-B, Pyramidal tract conduction abnormalities in the both lower extremity; SSEP, short latency somatosensory evoked potential; VEP, visual evoked potential; BAEP, brainstem auditory evoked potential; APPI-B, auditory pathway peripheral segment impairment-bilateral; APCI-R, auditory pathway central segment impairment-right.

To summaries the clinical characteristics of the family, four female and two male patients were evaluated. The main clinical symptoms were ataxia (5/6, 83.3%), insomnia (4/6, 66.7%), dysarthria (3/6, 50%), affective disorders (3/6, 50%), blurred vision (3/6, 50%), positive pathological signs (3/6, 50%), hearing loss (2/6, 33.3%), tongue muscle atrophy and muscle bundle tremor (1/6, 16.7%). (Table 1)

In electrophysiological examinations, two patients (II_5_ and II_11_) had central and peripheral damage to the auditory pathway as revealed by BAEP, which showed the disappearance of I–III waves and normal V waves or prolonged I–III or III–V interpeak latency. Two patients (II_5_ and II_11_) had hearing loss, as shown in the PTA test, mainly in the high-frequency hearing threshold. One patient (II_11_) had peripheral nerve damage Two patients (II_5_ and II_11_) had positive pyramidal tract sign by MEP (Supplementary Figure S1) However, all serum examination results were within the normal range.

### Neuroimaging results

**FIGURE 2:**
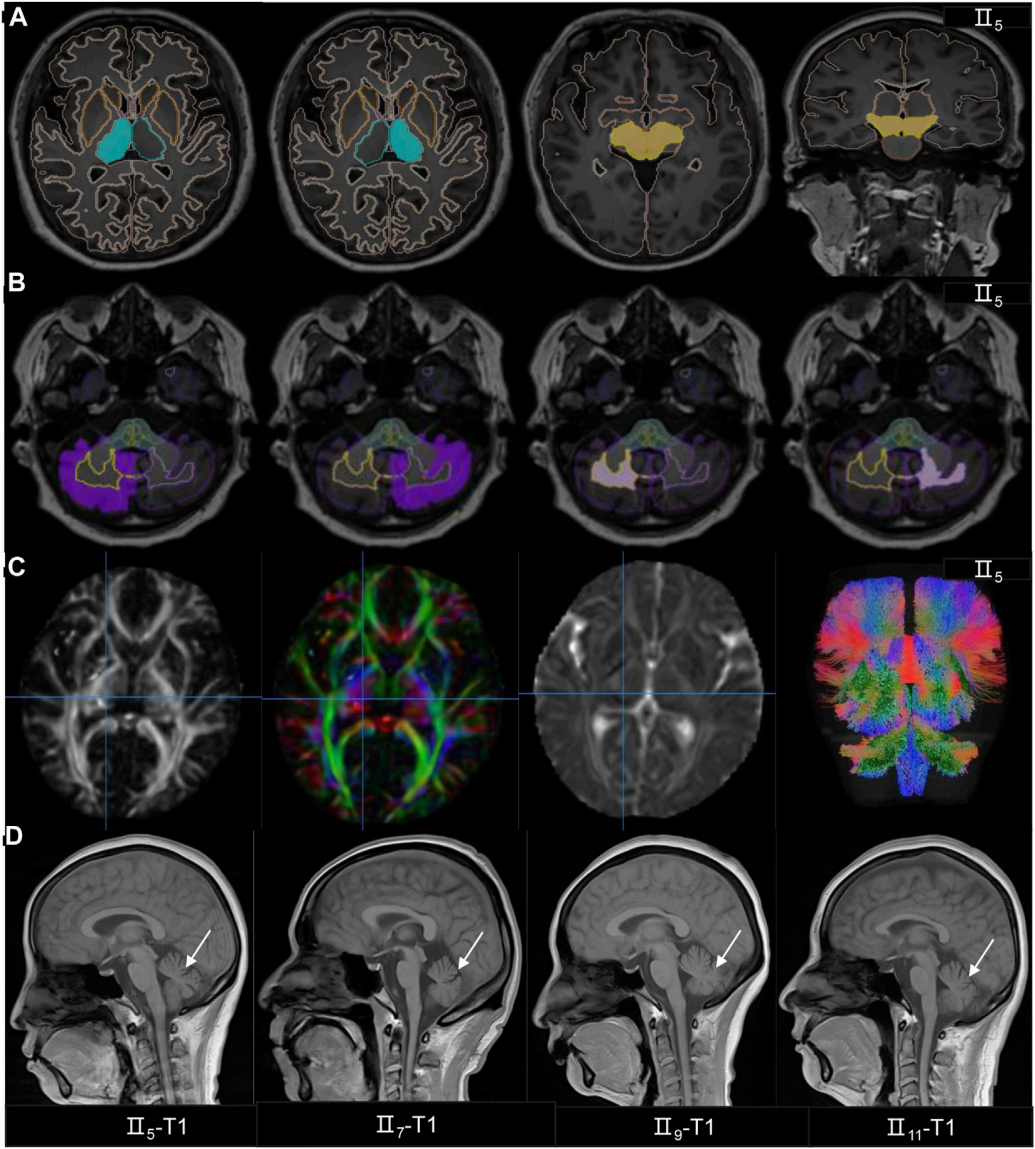
The MRI findings of the SCA family. **(A–B)** The 3D-T1 analysis showed the patient’s (II_5_) midbrain and thalamus volumes were normal, but the volumes of gray matter and white matter volumes in the cerebellum had decreased. **(C)** The patient’s (II_5_) DTI analysis showed the FA, DEC, AD and white matter bundles followed by fiber quantification. **(D)** MRI T1 images are in order II_5_,II_7_,II_9_ and II_11_, and T1 indicates atrophy of the cerebellum.

Two experienced imaging physicians interpreted and processed the images. They suggested that 3 patients (II_5,_ II_7_ and II_11_) had visual microcephaly, and II_9_ was assessed as normal (Figure 2D). 3D-T1 analysis showed that the patient had reduced cerebellar volume, less cerebellar white matter volume, and reduced pons volume than the controls (Figures 2A, B, Supplementary Table S1). DTI analysis also showed that the patient had reduced FA values in the superior and inferior peduncles of the left cerebellum compared to the controls (corrected *p* < 0.05) (Figure 2C; Supplementary Table S2).

## Genetic analysis

### SCA type determination

Because of the clinical characteristics of SCA in this family, genetic testing was used to determine the SCA subtype. The *NOP56* capillary electrophoresis map of the proband (II_5_) showed a single peak (Figure 1B), and a characteristic ladder pattern with a 6-bp periodicity on the electropherogram was identified by RP-PCR (Figure 1C). The results for the other affected members displayed the same features. We also found the abnormal CAG expansion repeat of the SCA17-associated *TBP* gene in this pedigree (Figure 1D), following the verification by Sanger sequencing that the CAG/CAA repeat number was 43. It is reported that most patients carry intermediate *TBP*_41-49_ alleles that show incomplete penetrance. Also, the parkinsonism, dystonia, seizure and chorea were typical features occurred in SCA17 can’t observed in this pedigree. A study showed that the ataxia-related phenotype occurs when *TBP* is in the incomplete penetrance genotype and also has *STUB1* variants. (Magri et al., 2022) However, we did not detect *STUB1* variants in the affected family members. Therefore, we considered that the aberrant CAG repeat expansion of the *TBP* in this family did not contribute to the clinical phenotype in this family. The results of RP-PCR are shown in (Supplementary Table S2). WES revealed no mutations in genes associated with ataxia in this pedigree.

### Long-read genome sequencing

**FIGURE 3:**
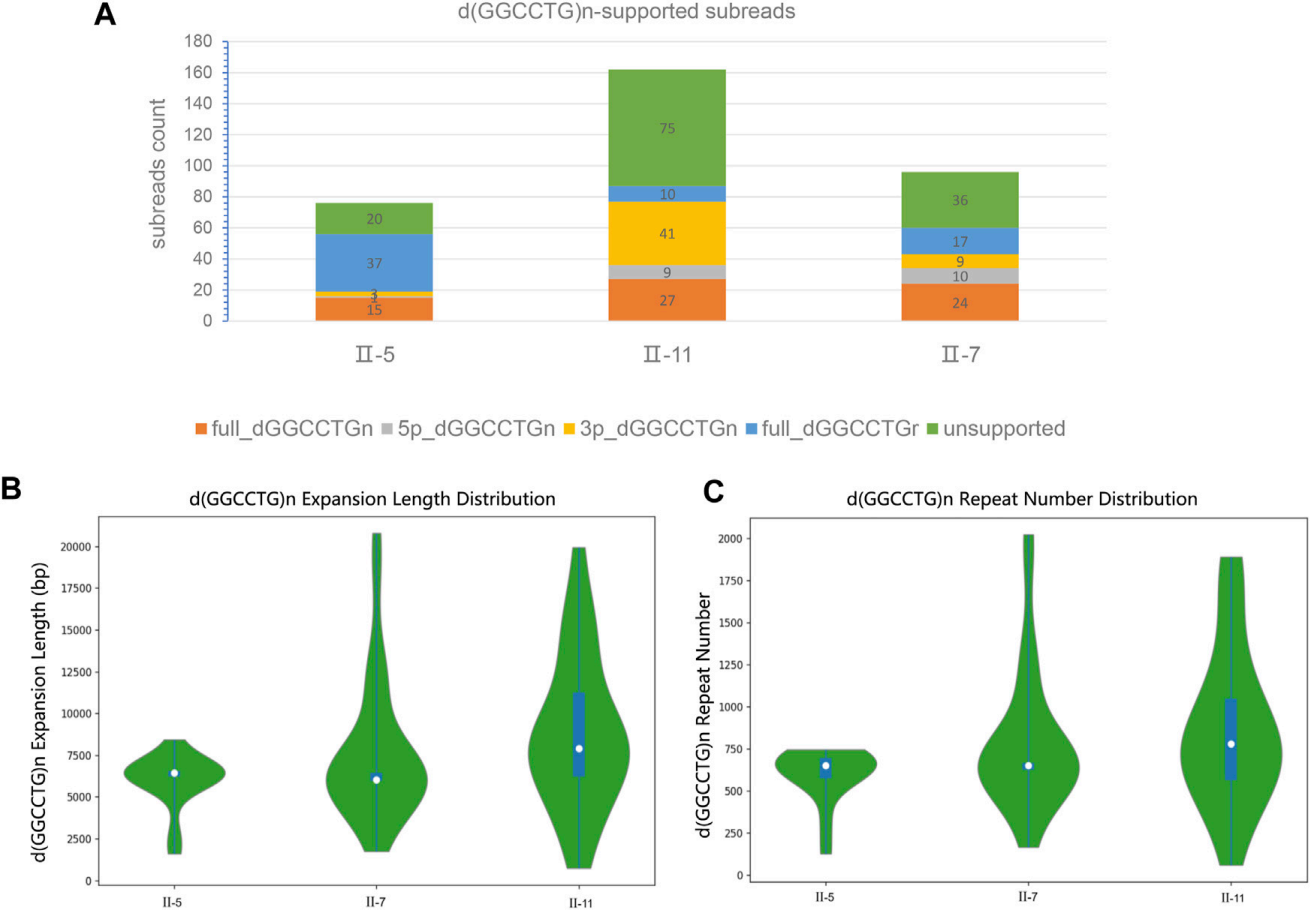
The classification and statistics of the subreads based on SMRT sequencing. **(A)** The numbers of classification d (GGCCTG) n-supported subreads. **(B)** The full_dGGCCTGn subreads of length distribution in three patients. **(C)** The full_dGGCCTGn subreads of repeat number distribution in three patients. These data suggests the existence of somatic heterogeneity in SCA36 patients.

The results of the SMRT sequencing data for the three samples are shown in (Supplementary Table S4). To avoid errors in SMRT sequencing technology and increase the reliability of sequencing sequences, the sequencing coverage was set to ×100, and the raw data reached more than 300 Gb. We focused on the structural variation analysis of the assembled genome for intron 1 of the *NOP56* gene and its sequences on both sides. The filtered subreads were then aligned to GRCh38 and fake GRCh38 reference genomes. Based on the comparison, subreads that were completely or partially across d (GGCCTG) n were extracted. The subreads were sorted into full_dGGCCTGn, 5p_GGCCTGn, 3p_GGCCTGn and full_dGGCCTGr, and the statistics of each subread were recorded (Table 2; Figure 3A). The GGCCTG motif was found in all three samples, and subreads with more than 650 repeats were counted in each sample (Table 2). The three samples had more than 650 subreads, indicating that all three carried pathogenic repeat variants and could be clearly diagnosed as SCA36.

**TABLE 2:** The statistics based on SMRT sequencing about the subreads.

Sample ID	Subread number	dGGCCTG 650+	dGGCCTG 650 + ratio	Full dGGCCTGn	Max length (bp)	Mean length (bp)	Min Length (bp)	Max number of repeats	Mean number of repeats	Min number of repeats
II-5	76	9	11.84	15	8,432	5,988	1,602	746	598	127
II-7	162	23	14.20	27	20,788	7,663	1747	2023	787	166
II-11	96	20	20.83	24	19,939	8,849	743	1890	868	60

dGGCCTG 650 + Ratio: The ratio of subreads with the GGCCTG of number repeats more than 650 to the total subreads.

We analyzed the genotype and phenotype in the patients. The full_dGGCCTGn subreads of three patients (II_5_, II_7_, and II_11_) were 15,27, and 24, respectively, and the length of each subread was different. The average (repeat region) length of II_5_ was 5,988 bp and the average repeat (unit) number was 598, the average length of II_7_ was 7,663 bp and the average number of repeats was 787; while the average length of II_11_ was 8,849 bp and the average number of repetitions was 868 (Figures 3B, C). The proportion of subreads with more than 650 repeat number was 11.84%, 14.20%, and 20.83% for II_5_, II_7_, and II_11_, respectively. SARA score was used to assess the severity of ataxia symptoms in patients. The scores of the three patients (II_5_, II_7_, and II_11_) were 10, 8.5, and 8.5, respectively. However, we considered that the disease duration of the three patients (II_5_, II_7_, and II_11_) is approximately 16, 10 and 7 years. From the perspective of disease progression, II_11_ had the fastest progression, followed by II_7_, and II_5_ the slowest. It is reported that the repeat size is associated with the clinical features in the disease with repeat expansion like Huntington’s disease. (Trottier et al., 1994) Combining genotype-phenotype analysis, we speculated that the average length, the average number of repeats of full_dGGCCTGn subreads, and the proportion of subreads with more than 650 repeats were associated with the age of onset and disease progression.

**FIGURE 4:**
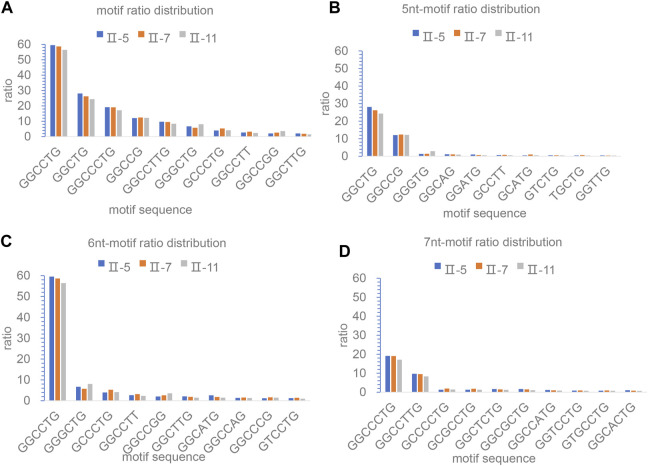
The analysis and illustration of the motifs based on the SMRT sequencing. The motif of all subreads ratio distribution in the region of repeat expansions. The ratio is calculated as in percent of the nucleotides of each motif divided by the total number of nucleotides for each expansion. The Top 10 motif **(A)**, Top 10 5 nt-motif **(B)**, 6 nt-motif **(C)** and 7 nt-motif **(D)** of all subreads ratio distributions in the region of repeat expansions.

The motif structure of long-read sequencing study in *NOP56* repeat expansion will be important to determine heterogeneity and whether the repeats are interrupted by non-GGCCTG content. Therefore, we further analyzed the composition and distribution of motifs within the repeat expansion regions of the three samples. The results showed that both the number and proportion of motifs were dominated by GGCCTG, and the rest were GGCTG, GGCCCTG, GGCCG, and GGCCTTG (Figure 4C). It was also found that the motifs mostly consisted of 5-nt, 6-nt, and 7-nt. It suggested that SCA36 patients had interruptions in the repeat expansion region, and the motifs varied around the core motif of GGCCTG. However, the TOP10 motifs, i.e., 5 nt, 6 nt, and 7 nt motifs, were not significantly different among the three samples (Figures 4B–D).

**FIGURE 5:**
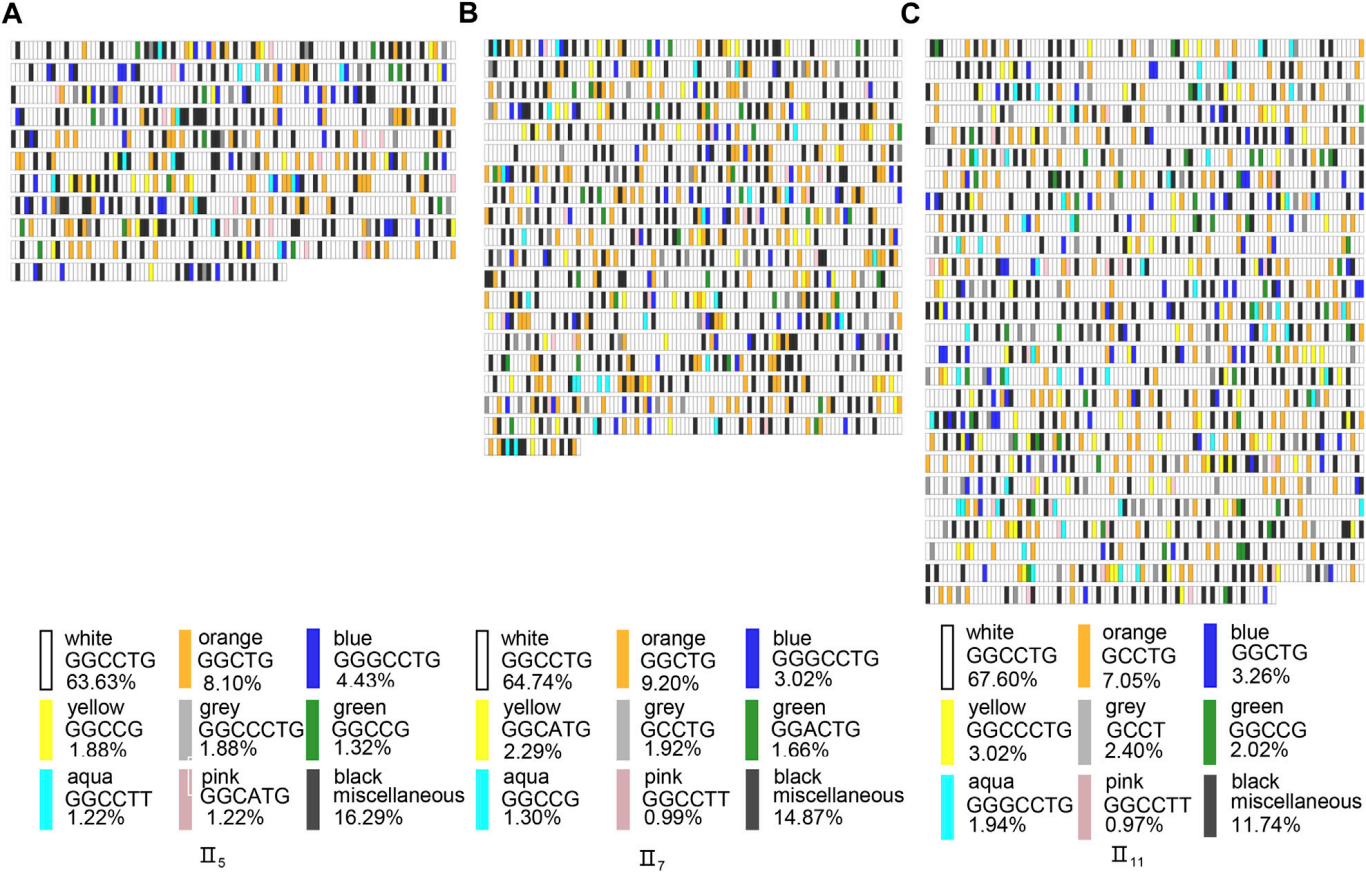
*NOP56* repeat expansion sequence schematics based on long-read sequencing with the highest GGCCTG repeat number full-length subreads of three patients. The schematics and expansion size shown are based on the highest GGCCTG number of repeats for the patients in the pedigree as follows: II_5_ **(A)**, II_7_ **(B)**, II_11_ **(C)**. The Rectangles represent sequence motifs, as indicated by the color key, in the 5’ (upper left) to 3’ (lower right) direction. Black rectangles indicated the motif is less than 1% of all the full-length subreads.

We performed IGV visualization analysis of each full-length subread of the three patients and produced correlation schematics based on the Practical Extraction and Report Language, which showed no obvious regularity in the interruptions. We selected the full-length schematics with the highest GGCCTG repeat number from each of the three patients for analysis (Figures 5A–C) The various motifs showed a free insertion pattern with no obvious regularity; therefore, we think that the position of the interruptions may not be clearly associated with the phenotype of the patients in this pedigree.

## Discussion

We confirmed the diagnosis of a Han Chinese SCA36 pedigree by combining RP-PCR, WES, and SMRT genetic testing techniques. The main clinical features of this pedigree are late-onset ataxia symptoms, with a presymptomatic presence of affective and sleep disorders. The disease has been reported in the literature to have a predominance of late-onset ataxia and motor neuron disease. Hearing loss, blurred vision, and positive pathological reflexes have also been reported. (Kobayashi, et al., 2011; Garcia-Murias, et al., 2012; Ikeda, et al., 2012; Sugihara, et al., 2012) The family we collected not only exhibited some features consistent with previous reports, but patients also had sleep disorders in the presymptomatic period, which have not been previously reported. In addition, sleep was influenced by affective disorder, but II_9_ who showed no affective disorder symptoms still had sleep disorders, suggesting that sleep disorders are related to the disease.

For imaging, we applied 3D-T1-based brain volume segmentation measurements and DTI-based brain white matter fiber tracking techniques for the brain analysis of a SCA36 patient and found that the patient had reduced cerebellar volume, less cerebellar white matter volume, and reduced pons volume. Also, the patient’s left cerebellar superior and inferior peduncle FA values were reduced, and SCA36, cerebellum, and pons structures in the brain cadre were also affected. The presence of wake-promoting centers in the pons site includes noradrenergic neurons in the locus coeruleus and serotonergic neurons in the dorsal raphe nuclei. (Xu et al., 2015) Considering that our patient had chronic insomnia in the presymptomatic period, this may be related to the reduced volume of the pons affecting the function of the nerve nuclei.

We performed long-read genome sequencing of three patients in the pedigree and analyzed the sequencing results. The full-length subreads of the three patients were 15, 27, and 24, respectively, and the length of each subread and the repeat time of GGCCTG were different. It suggested that somatic heterogeneity exist in our SCA36 patients’ samples. Although blood samples were tested, it is speculated that there is a similar phenomenon in the affected tissues. Previous studies have shown that the somatic heterogeneity of the repeat sizes is quite common in similar repeat expansion disorders, such as ALS/FTD caused by the *C9orf72* and SCA10 caused by the *ATXN10*. (Matsuura et al., 2004; Ebbert et al., 2018) However, the somatic heterogeneity of SCA36 has only been mentioned in the literature and has not been described in detail. (Lopez and He 2022) We firstly verified and described the phenomenon from the perspective of sequencing data, which can reflect somatic mutations definitely. (Breuss et al., 2022; Cagan et al., 2022; Miller et al., 2022)

Studies have shown that repeat expansion diseases are characterized by genetic anticipation, followed by the earlier age of onset (AOO) and more severe symptoms in subsequent generations. (Carpenter 1994) Otha et al. (2020) reported a four-generation SCA36 family pedigree that showed longer repeat length and earlier age of onset of disease from one generation to the next, but due to technical limitations, only the overall length of the repeats could be measured, and no measurement of repeat number could be made. Since the mother of the proband in the family we reported has died and the patient’s offspring have not yet developed the disease, it is not possible to prove genetic anticipation. From the observations of the siblings and analysis of sequencing in this SCA36 pedigree, it suggested that the mean repeat length and time were same changing trend with age at onset and opposite changing with disease severity. However, the sample size was small, and more SCA36 pedigrees are needed to validate our results.

It has been shown that interruptions exist in similar nucleotide repeat expansion diseases and are associated with the disease course and phenotypes, for example, the seizure symptoms of SCA10 patients correlated with the position of interruptions in the ATTCC motif, and the interruption of CAA repeat number in SCA2 correlated with the severity in patients. (Pulst et al., 1996; McFarland et al., 2014) Therefore, we obtained full-length subreads using long-read genome sequencing to analyze the interruption motifs. In addition, because indels are the most common errors in SMRT sequencing, we set the coverage to 100x to reduce the error to less than 0.01%. We performed IGV visualization of all full lengths and depicted motif content of >1% schematically for motif structure analysis. The results show that interruptions are found in these regions, but the insertions of the interruptions were random and had no obvious regularity. We also found that the motifs were all 5 nt, 6 nt, and 7 nt, and the motifs were always transformed around the core motif GGCCTG. However, the patients in this family did not show any interruptions correlated with their clinical features.

To date, researchers have relied on Southern blotting to measure the GGCCTG repeat number of SCA36 pathogenic sequencing, but its technical limitations prevented us from obtaining specific repeat sequences. Therefore, we attempted to use third-generation sequencing technology to address diagnostic questions as well as to determine the prognosis. Oxford Nanopore Technologies (ONT) sequencing technology features long-read length but high error rate. (Ebbert, et al., 2018) Therefore, we used SMRT sequencing technology based on the PacBio sequel II platform, which has a long-read length, high accuracy, and no GC preference. Initially, we applied third-generation targeting technology which was widely used for the HLA typing to sequence the pathogenic region of *NOP56*, however, it was unsuccessful because of the complexity of the repeat expansion region. Then we sequenced and assembled the whole genome, focusing on the *NOP56*. The results showed that SMRT sequencing could complete the sequencing of repeat expansion regions with high GC content and also found the presence and location of structural variants that could describe the composition of specific repeat motifs, which bodes well for the promising application of this technology in similar diseases.

In this study, we applied the PacBio platform for the first time to perform complete sequencing of the pathogenic region of the genome of SCA36 patients, which is important for revealing the genetics of the clinical phenotype. First, we found that the repeat region of *NOP56* was not a simple GGCCTG hexanucleotide motif as there were interruptions in region, and the interruptions always varied around the core motif GGCCTG. Second, the correlation between the number of repeats, age of onset and severity was revealed. Last, we further validated and elaborated on the existence of somatic heterogeneity in SCA36. Thus, we believe this study is of great significance as it further revealed the pathogenesis of the disease and lays a theoretical foundation for the study of its pathogenesis.

The present study has some limitations. First, our sample size was small, and the study population consisted of only one family with similar symptoms. Second, we used blood to verify somatic heterogeneity and can´t obtain the cerebellar tissue for further validation. Third, there are two main types of pathogenesis regarding SCA36, one for the gain-of-function hypothesis of repeat-containing RNAs, where RNA transcripts of repeat expansion regions accumulate and sequester key RNA-binding proteins, leading to neuronal dysfunction. (Zu et al., 2011) The other is non-ATG-driven (RAN) translation in SCA36, the expanded GGCCTG repeats can be transcribed from both directions, producing sense and antisense transcripts, and the RAN translation results in different dipeptide repeats (DPRs), and these DPRs can elicit cytotoxicity in various ways, leading to neurological damage. (Todd et al., 2020) A single 5 nt or 7 nt motif interruption could cause a frameshift, resulting in translational transitions from the relatively DRPs, potentially affecting repeat-associated non-ATG (RAN) translation and disease development and progression. However, we did not perform relevant experiments to verify these assumptions. Furthermore, we plan to study these three aspects in depth. In addition, the current PacBio platform sequencing is expensive, and its application in clinical disease diagnosis is limited. In the future, we will continuously improve and optimize the process, reduce the sequencing cost, perform long read-length sequencing of the repeat expansion region of *NOP56* in more SCA36 patients, and further investigate the relationship between the repeat expansion region and phenotypes, which will greatly promote the speed of SCA36 pathogenesis mechanism research and drug development.

## Data Availability

The data presented in the study are deposited in the NCBI Sequence Read Archive (SRA) repository, accession number PRJNA918832.
