# Supplementary figures and images for: A Chinese SCA36 pedigree analysis of NOP56 expansion region based on long-read sequencing
Source: Front Genet. 2023 Mar 27;14:1110307. doi: 10.3389/fgene.2023.1110307 (PMC10083286; doi:10.3389/fgene.2023.1110307)

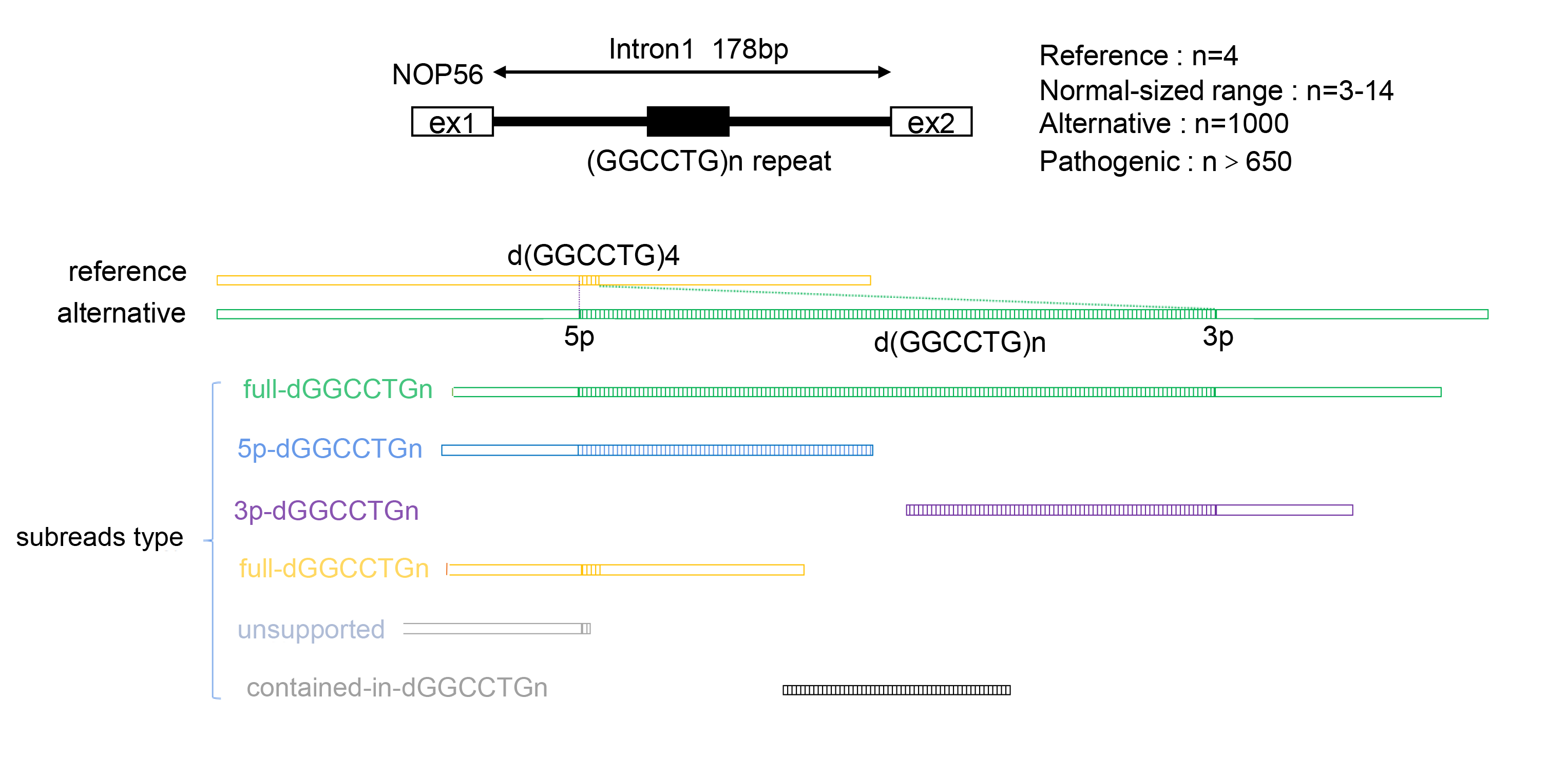

Supplement: Supplementary file 1 [file Image2.TIF]

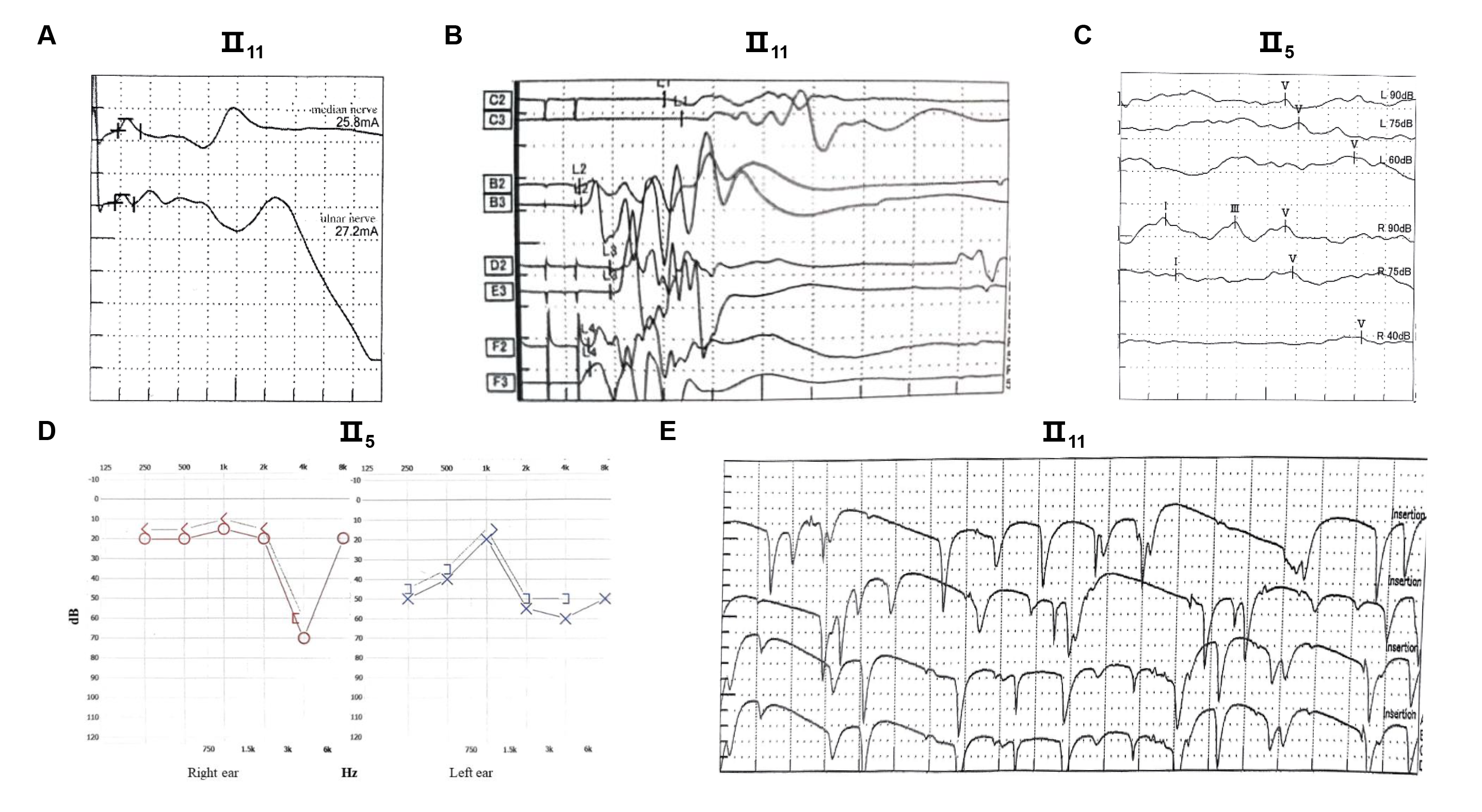

Supplement: Supplementary file 2 [file Image1.TIF]
