# Supplementary material for: A Chinese SCA36 pedigree analysis of NOP56 expansion region based on long-read sequencing
Source: Front Genet. 2023 Mar 27;14:1110307. doi: 10.3389/fgene.2023.1110307 (PMC10083286; doi:10.3389/fgene.2023.1110307)
Supplement: Supplementary file 3 [file DataSheet1.docx]

**Supplement table 1：Brain regions with reduced volumes**

| Brain Regions | Ⅱ-5 | | | Ⅱ-9 | | | Ⅱ-11 | | | | Ⅱ-15 | | |
| --- | --- | --- | --- | --- | --- | --- | --- | --- | --- | --- | --- | --- | --- |
|  | Volume  （cm^3^） | % Vol | normal range | Volume  （cm^3^） | % Vol | normal range | | Volume  （cm^3^） | % Vol | normal range | Volume  （cm^3^） | % Vol | normal range |
|  |  |  |  |  |  |  |  |  |  |  |  |  |  |
| Cerebellum  White matter | 13.43 | **0.95** | 1.25-1.82 | 14.81 | **1.19** | 1.27-1.75 | | 14.12 | **1.19** | 1.30-1.82 | 20.93 | 1.39 | 1.27-1.78 |
| Cerebellum  Gray matter | 65.04 | 4.62 | 4.61-6.33 | 73.46 | 5.93 | 4.70-6.22 | | 56.91 | 4.81 | 4.76-6.42 | 89.73 | 5.99 | 4.79-6.28 |
| Cerebellum | 78.47 | **5.57** | 5.85 - 8.13 | 88.27 | 7.12 | 5.97-7.97 | | 71.03 | **6.00** | 6.05-8.23 | 110.66 | 7.38 | 6.06-8.05 |
| Thalamus | 10.82 | 0.77 | 0.50-0.81 | 9.82 | 0.79 | 0.47-0.84 | | 9.93 | 0.84 | 0.51-0.85 | 9.54 | 0.64 | 0.49-0.81 |
| Midbrain | 9.17 | 0.65 | 0.53-0.80 | 8.56 | 0.69 | 0.55-0.81 | | 7.57 | 0.64 | 0.55-0.83 | 9.54 | 0.64 | 0.56-0.80 |
| Pons | 11.84 | **0.84** | 0.87-1.20 | 11.03 | **0.86** | 0.88-1.16 | | 9.94 | **0.84** | 0.88-1.16 | 14.24 | 0.95 | 0.90-1.20 |
| Medulla | 4.37 | 0.31 | 0.25-0.49 | 3.84 | 0.31 | 0.28-0.45 | | 3.67 | 0.31 | 0.28-0.48 | 4.50 | 0.30 | 0.30-0.48 |

**Supplement table 2：Brain regions with reduced FA values in patients**

| fiber bundle | Patients（mean±SD） | Controls（mean±SD） | P-value |
| --- | --- | --- | --- |
| Corticospinal tract-left | 0.544±0.010 | 0.549±0.029 | 0.786 |
| Corticospinal tract-right | 0.534±0.008 | 0.545±0.019 | 0.394 |
| Inferior cerebellar peduncle-left | 0.390±0.009 | 0.417±0.013 | 0.012 |
| Inferior cerebellar peduncle-right | 0.354±0.022 | 0.395±0.034 | 0.093 |
| Middle cerebellar peduncle | 0.464±0.002 | 0.468±0.024 | 0.781 |
| Optic radiation-left | 0.479±0.012 | 0.453±0.023 | 0.108 |
| Optic radiation-right | 0.488±0.008 | 0.458±0.029 | 0.125 |
| Superior cerebellar peduncle-left | 0.437±0.009 | 0.495±0.009 | 0.002 |
| Superior cerebellar peduncle-right | 0.436±0.013 | 0.456±0.015 | 0.066 |
| Corpus Callosum - all | 0.466±0.015 | 0.450±0.017 | 0.212 |

**Supplement table 3：The TP-PCR result in this SCA pedigree**

| Sample ID | CAG/CAA repeat in TBP | GGCCTG repeat in NOP56 |
| --- | --- | --- |
| Ⅱ-5 | (34,35) | **((9,>15)** |
| Ⅱ-7 | **(34,43)** | **(9,>15)** |
| Ⅱ-9 | (34,35) | **(9,>15)** |
| Ⅱ-11 | **(34,43)** | **(9,>15)** |
| Ⅱ-13 | **(34,43)** | **(9,>15)** |
| Ⅱ-15 | **(34,43)** | (9,13) |
| Ⅲ-4 | (34,35) | **((9,>15)** |
| Ⅲ-10 | **(34,43)** | **(9,>15)** |

**Supplement table 4：Based on SMRT sequencing technology generating Data and** **Assembled Individual Genome Contig Sequence Statistics Table**

| Sample ID |  | Subreads |  | | Contig | | | | | | Coverage |
| --- | --- | --- | --- | --- | --- | --- | --- | --- | --- | --- | --- |
|  | Mean  Length（Kb） | N50（Kb） | Read Base（Gb） | Longest（bp） | | N50(bp) | L50 | Length  (bp) | Number | |  |
| Ⅱ-5 | 19.52 | 24.80 | 312.05 | 23708960 | | 11830687 | 68 | 2816755886 | | 801 | 100x |
| Ⅱ-7 | 20.67 | 24.32 | 304.74 | 27553795 | | 4124687 | 181 | 2787269189 | | 1868 | 100x |
| Ⅱ-11 | 16.36 | 24.16 | 324.31 | 76221884 | | 13316911 | 60 | 2811595655 | | 726 | 100x |

Subreads Mean Length(Kb)：Average length of Subreads after removing linkers ;Subreads N50(Kb)：N50 of Subreads after removing the linker; Subreads Read Base (Gb): The total number of bases after removing the linker

N50: The assembled contig sequences are arranged from long to short and accumulated, and the length of the contig when the accumulated length reaches 50% of the total length. Assembly continuity evaluation index, the larger the more continuous; L50: The assembled contig sequences are arranged from long to short and accumulated, and the sequence number of the contig when the accumulated length reaches 50% of the total length. Assembly continuity evaluation index, the smaller the more continuous; Length(bp): The total length of contig obtained by assembly;

Number: The number of contigs obtained by assembly.
